# Supplementary material for: Partial Versus Total Omentectomy in Patients with Gastric Cancer: A Systemic Review and Meta-Analysis
Source: Cancers (Basel). 2021 Oct 3;13(19):4971. doi: 10.3390/cancers13194971 (PMC8508137; doi:10.3390/cancers13194971)
Supplement: Supplementary file 1 [file cancers-13-04971-s001.zip › supplementary figures.pdf]

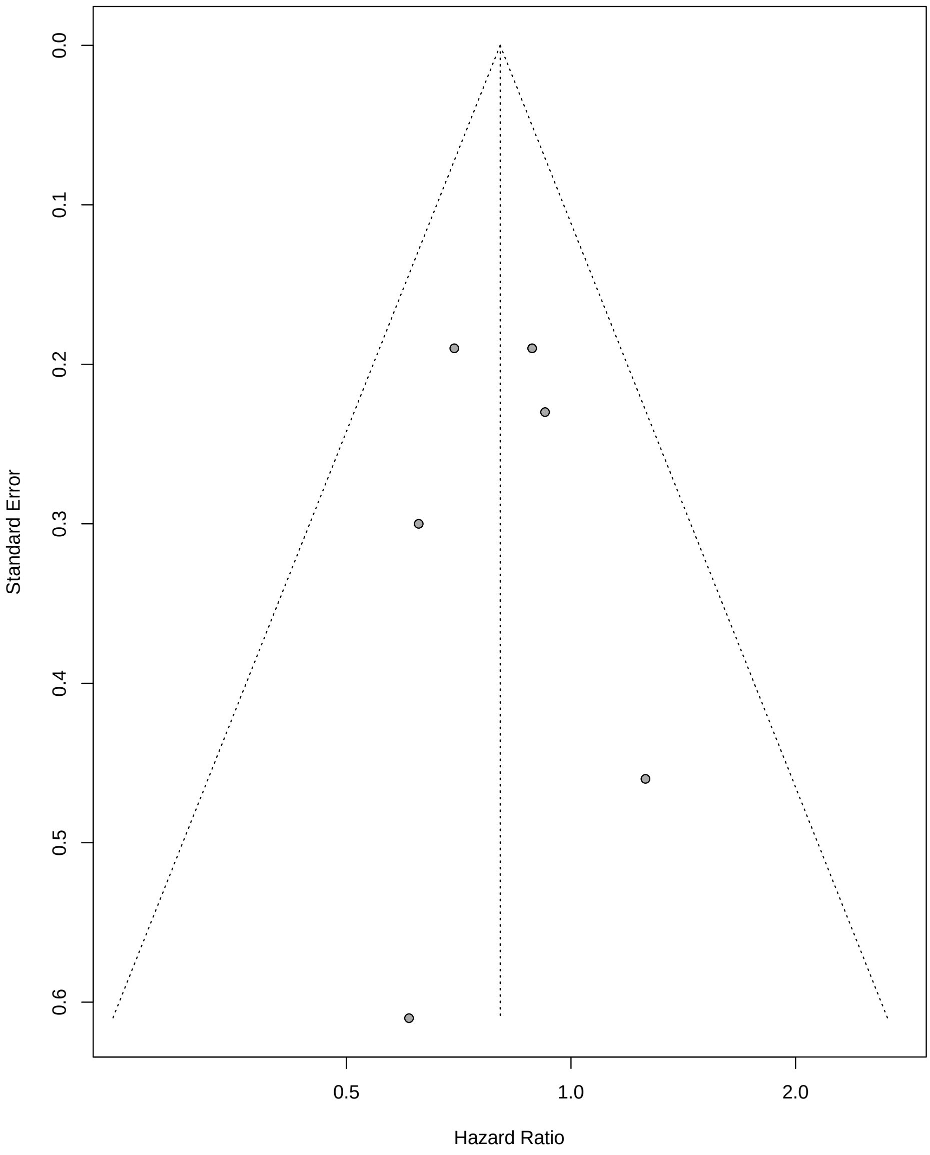

Figure S1. Funnel plot of OS between the partial omentectomy and the total omentectomy groups.

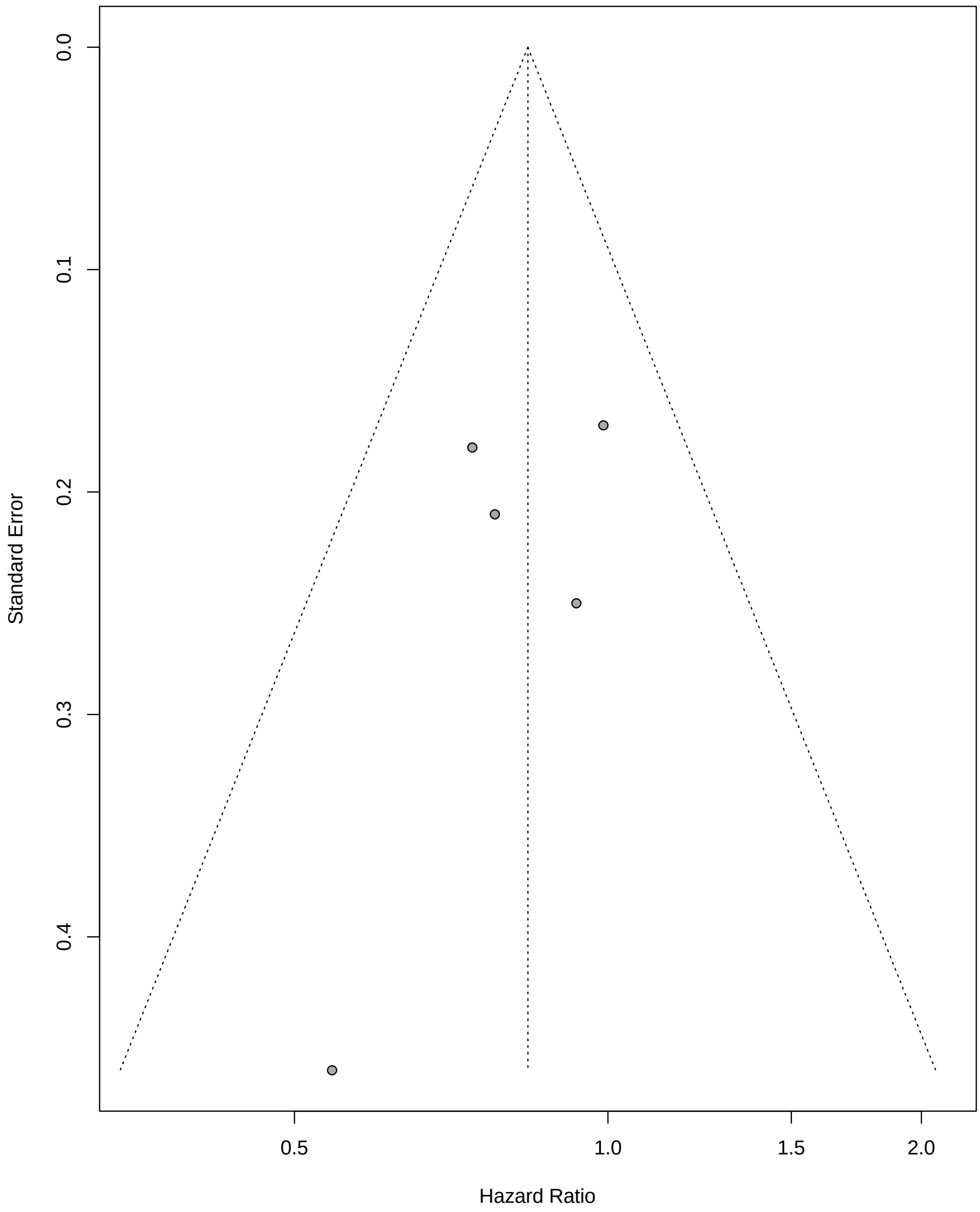

Figure S2. Funnel plot of DFS between the partial omentectomy and the total omentectomy groups.

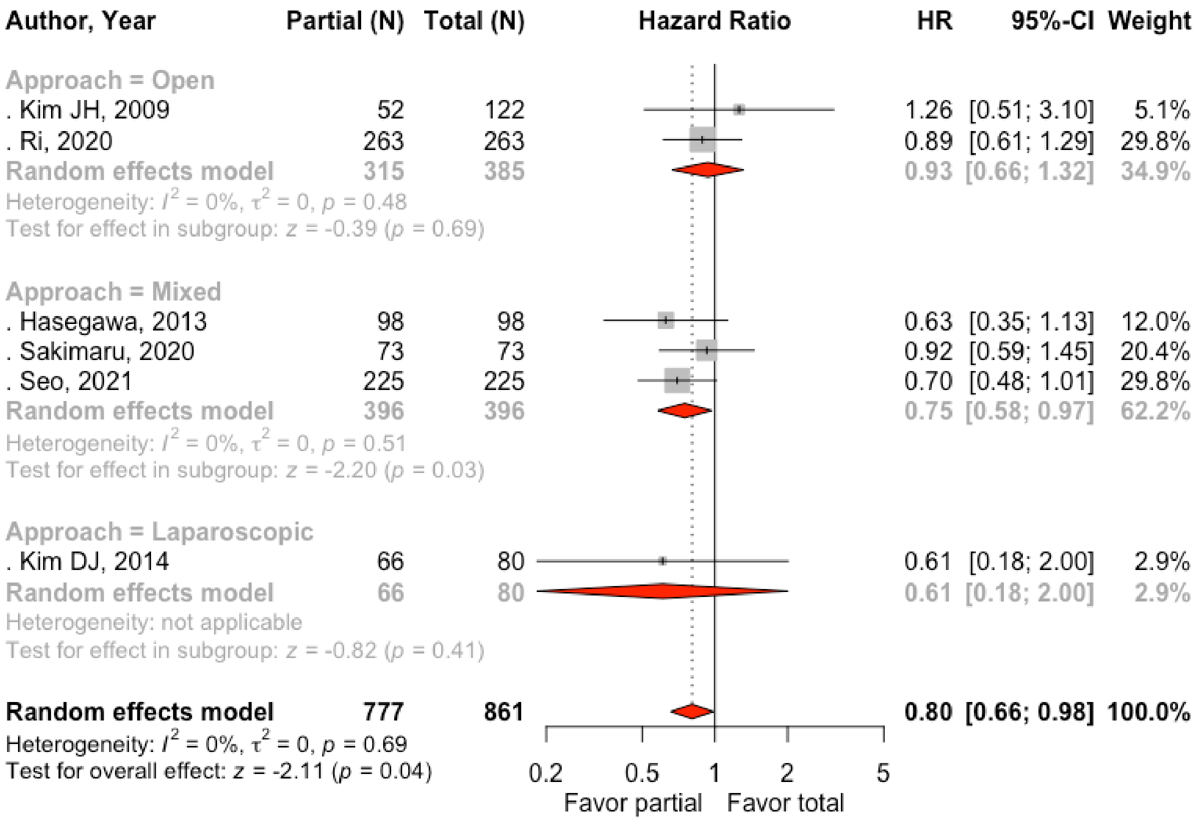

Figure S3. Forest plot comparing the overall survival between the partial omentectomy and the total omentectomy groups stratified by surgical approaches.

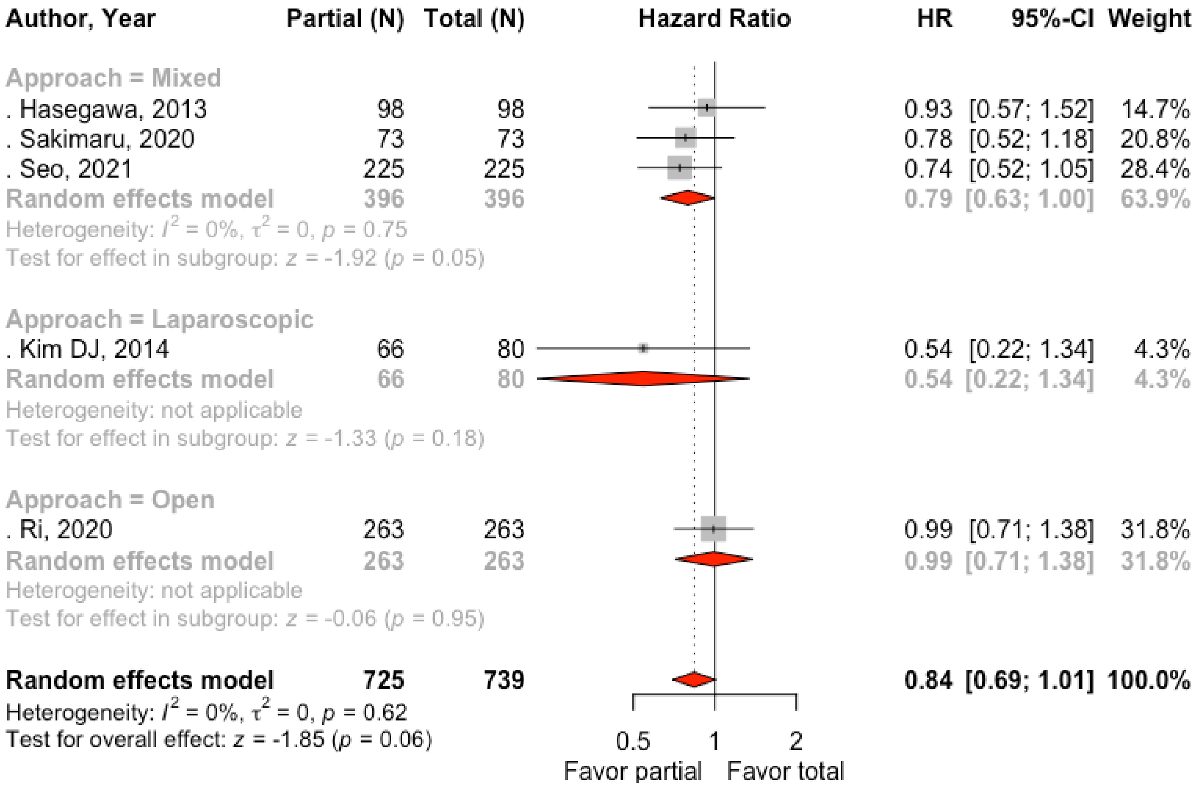

Figure S4. Forest plot comparing the disease-free survival between the partial omentectomy and the total omentectomy groups stratified by surgical approaches.
